# Unpacking the enabling factors for hand, cord and birth-surface hygiene in Zanzibar maternity units

**DOI:** 10.1093/heapol/czx081

**Published:** 2017-07-12

**Authors:** Giorgia Gon, Said M Ali, Catriona Towriss, Catherine Kahabuka, Ali O Ali, Sue Cavill, Mohammed Dahoma, Sally Faulkner, Haji S Haji, Ibrahim Kabole, Emma Morrison, Rukaiya M Said, Amour Tajo, Yael Velleman, Susannah L Woodd, and Wendy J Graham

**Affiliations:** 1 Department of Infectious Disease Epidemiology, The London School of Hygiene and Tropical Medicine, Keppel Street, London; 2 The Soapbox Collaborative, Keppel Street, London; 3 Public Health Laboratory - Ivo de Carneri, Wawi, Chake Chake, Pemba, Zanzibar, Tanzania; 4 Centre for Actuarial Research, University of Cape Town, Private Bag, Rondebosch, South Africa; 5 CSK Research Solutions, Sinza B, Mori Street, Dar Es Salaam, Tanzania; 6 The Ministry of Health of the Revolutionary Government of Zanzibar, Maternal and Child Health Office, Ministry of Health Zanzibar, Zanzibar, Tanzania; 7 WaterAid, 27 Cranbrook Drive, Maidenhead, Berkshire; 8 The University of Sheffield, 2 Sudan Avenue, Brackley, Northamptonshire, UK; 9 WaterAid, Dar Es Salaam, Tanzania and; 10 WaterAid, 47-49 Durham Street, London, UK

**Keywords:** Maternal and child health, prevention, health care, health behaviour, water

## Abstract

Recent national surveys in The United Republic of Tanzania have revealed poor standards of hygiene at birth in facilities. As more women opt for institutional delivery, improving basic hygiene becomes an essential part of preventative strategies for reducing puerperal and newborn sepsis. Our collaborative research in Zanzibar provides an in-depth picture of the state of hygiene on maternity wards to inform action. Hygiene was assessed in 2014 across all 37 facilities with a maternity unit in Zanzibar. We used a mixed methods approach, including structured and semi-structured interviews, and environmental microbiology. Data were analysed according to the WHO ‘cleans’ framework, focusing on the fundamental practices for prevention of newborn and maternal sepsis. For each ‘clean’ we explored the following enabling factors: knowledge, infrastructure (including equipment), staffing levels and policies. Composite indices were constructed for the enabling factors of the ‘cleans’ from the quantitative data: clean hands, cord cutting, and birth surface*.* Results from the qualitative tools were used to complement this information.

Only 49% of facilities had the ‘infrastructural’ requirements to enable ‘clean hands’, with the availability of constant running water particularly lacking. Less than half (46%) of facilities met the ‘knowledge’ requirements for ensuring a ‘clean delivery surface’; six out of seven facilities had birthing surfaces that tested positive for multiple potential pathogens. Almost two thirds of facilities met the ‘infrastructure (equipment) requirement’ for ‘clean cord’; however, disposable cord clamps being frequently out of stock, often resulted in the use of non-sterile thread made of fabric. This mixed methods approach, and the analytical framework based on the WHO ‘cleans’ and the enabling factors, yielded practical information of direct relevance to action at local and ministerial levels. The same approach could be applied to collect and analyse data on infection prevention from maternity units in other contexts.

**Key Messages**

- In the context of maternity units in Zanzibar, we found substantial gaps in coverage of key determinants of infection prevention practices essential at the time of birth. In particular areas for further improvement include knowledge and training, and infrastructure.
- This is the first study based on an analytical approach using both mixed methods and a combination of two sets of WHO guidelines: (i) WHO ‘cleans’ necessary to ensure a clean birth; and (ii) WHO guidelines on the determinants of infection prevention practices. This novel approach yielded information of direct relevance to action at both local and ministerial levels, which we refer to as ‘actionable information’.
- This study’s analytical approach is applicable to other contexts when collecting and analysing data on infection, prevention and control from maternity units.

## Introduction

Worldwide estimates indicate 2.6 million possible cases of severe bacterial infections among newborns in 2012 across Sub Saharan Africa alone (Seale *et al.* 2014). Additionally, puerperal sepsis is estimated to occur in 4% of live births (AbouZahr 2003). Gordon, Semmelweiss, and Wendell-Holmes established the link between puerperal sepsis and poor hygiene at birth over two centuries ago (Gordon, 1795; Semmelweis, 1983; Gould, 2010), and it has been estimated that a clean birth in a facility could prevent 38% of newborn tetanus mortality (Blencowe *et al.* 2011).

A list of important clean birth practices (for example clean hands), was presented by the World Health Organization (WHO) in the ‘cleans’ framework (Blencowe *et al.* 2011). For the clean practices to be carried out, the necessary enabling environment needs to be in place. This falls under the broader umbrella of infection prevention and control practices (IPC). The new WHO guidelines on IPC in facilities identified core components required to improve IPC practices and ultimately reduce healthcare associated infections (WHO 2016), e.g. ensuring access to the relevant infrastructure such as safe water and sanitation (WHO 2009) or sterilization of key equipment.

There are few data on the performance of the clean practices around birth or on the status of the enabling environment necessary for the clean practices, apart from some emerging efforts on water and sanitation, including by the Joint Monitoring Program for Water Supply and Sanitation (WHO and UNICEF 2016). The need to develop indicators and to incorporate water and sanitation and hygiene (WASH) in routine health monitoring systems was recently emphasized in the *Call to Action* paper on WASH and maternal and newborn health and the WHO report on the issue (Velleman *et al.* 2014; WHO 2015).

We have two aims in this article. The first is to illustrate how the WHO cleans framework and a framework of enabling factors from the WHO IPC guidelines were used to produce actionable information to enable the Zanzibar Ministry of Health (MoH) to identify priorities to improve hygiene in their maternity units. The second is to present the main assessment findings, which examined the enabling factors of key ‘clean’ practices, including hands, cord and birth surface hygiene, in maternity units in Zanzibar. The data were collected during an assessment across maternity units in Zanzibar, commissioned by the MoH in 2013 to inform a quality improvement process for maternity wards.

The Revolutionary Government of Zanzibar is a semi-autonomous region of Tanzania; it is home to a population of about 1.3 million people spread over two main and several small islands, and has an independent MoH. As in mainland Tanzania, only 50% of births in Zanzibar occur in facilities, and great efforts in the last decade have reduced the maternal mortality ratio from 473 per 100 000 live births in 2006 to a ratio of 310 in 2013 (Zanzibar Annual Health Bulletin - 8th publication 2014). A modest increase in facility births in Tanzania, from 43.5 to 50.1%, between 1999 and 2010,(ICF International), along with the aim of the government to encourage all women to deliver in facilities, emphasizes the importance of making hygiene in maternity units a priority, and the opportunity this provides to prevent infections. Recent publications highlight the poor WASH environment where women give birth in The United Republic of Tanzania, both in facilities and at home (Shamba *et al.* 2013; Benova *et al.* 2014). Only 24% of delivery rooms have basic improved water and sanitation standards across a representative sample of facilities in Tanzania (Benova *et al.* 2014).

## Methods

Our first aim was to produce actionable information, meaning information that (1) is organized by the WHO ‘clean’ practices necessary to reduce maternal and newborn infection acquired at the time of delivery; (2) clearly identifies the behavioural factors from the WHO IPC guidelines that enable these clean practices and that can be addressed through MoH interventions; and (3) allows the root causes of the IPC gaps to be identified, using a mixed methods approach. We investigated four out of the six ‘cleans’: clean hands, clean cord (clamping and cutting), and a clean birth surface. The clean perineum of the mother at birth was excluded because of the weak evidence base for this clean (Blencowe *et al.* 2011) and the postpartum skincare of the newborn was excluded because we were focused on intrapartum care for data collection

The WHO IPC guidelines for facilities identified eight core components.(WHO 2016) We collected data in Zanzibar that allowed us to investigate four of these components that we refer to as behavioural factors in relation each of the four cleans we chose to investigate.

These enabling factors and their definition in this paper are:

- Knowledge and training (from WHO core component number 3)*—*what it is necessary to know to practice relevant IPC behaviour, including awareness of key practices and levels of training.
- Infrastructure (from WHO core component number 8)—the availability, access and maintenance of the infrastructure (e.g. water supply) and equipment required to perform the cleans.
- Staffing levels (from WHO core component number 7)—the presence of an adequate number of staff responsible for the relevant clean practice; health orderlies to clean the delivery surface; and skilled birth attendants (SBAs) for performing clean hands and clean cord. If no SBA is present, it is possible that the delivery will be carried out by an unqualified member of staff without any formal training on these cleans. In Zanzibar, the following cadres, who have between 2 and 8 years of professional training, are considered qualified to assist a birth: Nurse midwife, Public Health Nurse B, Maternal and Child Health Aid, Clinical officers, Assistant Medical Officers, Medical officers, and Obstetricians.
- Policies (from WHO core component number 2)—whether there are existing policies, guidelines or other indications (e.g. through posters) to prescribe the clean practice of interest. Information on policies was collected for all cleans except cord care.

### Data collection tools using a mixed methods approach

Three tool sets were used during the assessment: (1) a structured facility questionnaire, administered to the maternity in-charge or equivalent at the time of the interview in all facilities providing delivery services (*n* = 37), (2) a ‘walkthrough’ tool set (described below) and (3) semi-structured interviews conducted in a purposively selected sample of facilities in Zanzibar (*n* = 7). The seven facilities were selected by the Zanzibar MoH to represent the variation in facility type, volume of deliveries, location and levels of service quality. The tools described below were based on the WASH & CLEAN toolkit, adapted with the collaboration of key MoH stakeholders and administered in Swahili. The toolkit, previously used in India, Bangladesh and the Gambia, was developed by the Soapbox Collaborative from existing tools from international organizations to assess IPC on maternity units and is publically available online (Cross *et al.* 2016). The facility questionnaire was initially piloted in five facilities, and the walkthrough tools and the semi-structured interviews were piloted in four.

The tools were administered between 19 May and 10 September 2014. We conducted 26 semi-structured interviews with healthcare staff including in-charges (7), care providers in the maternity (7), orderlies (7) and maintenance staff (5) present in the facility at the time of the visit. One member per cadre per facility was invited to be interviewed. Staff selection was based on who was available at the time. The facility questionnaire and the semi-structured interviews focused on guidelines, training and infrastructure for IPC, WASH and solid waste management; barriers to maintaining good practice; and the actions needed to overcome them. Qualitative interviews were also conducted with 20 women attending vaccination services for their newborns at the seven facilities, who had delivered within the past 8 weeks. The team aimed to interview a minimum of two women at each facility visited; one who delivered at the facility under assessment and one who delivered at home but who was living around the facility catchment area. The first woman presenting in the relevant facilities during the assessment period who consented to participate in the study was interviewed. These interviews sought to capture women’s perception of an appropriate delivery environment, and their experiences during their most recent childbirth, particularly in relation to hygiene at the delivery unit. Interviews were conducted in Swahili and were tape recorded.

Two types of data were collected with the walkthrough tool set: (1) observations recorded in the walkthrough checklist, noting the availability and conditions of specific areas and equipment (e.g. labour ward room, toilets and cleaning equipment); and (2) microbiological samples taken using swabs of high-risk hand touch sites such as bedside lockers, delivery beds, cleaning equipment, and of water used for hand washing in the maternity unit. See Supplementary Material S1 for more details on the water sampling and microbiological swabs.

### Constructing indices for the enabling factors of the four ‘cleans’

For each ‘clean’ we built a composite index, using the facility questionnaire data (*n* = 37), that aimed to be represent each of the four enabling factors investigated: ‘knowledge and training, infrastructure, staffing levels’ and ‘policies’*.* The choice of index components was informed by published IPC international guidelines for each topic (EngenderHealth 2003, 2011; WHO 2015). This allowed us to standardise the analysis of the ‘cleans’’ enabling factors with relevant data from the facility questionnaire.

Table 1 describes the information used to build these indices. For the ‘knowledge and training’ index, we used questions that explored the topics discussed during IPC training received in the past year and questions around maternal and newborn care practices. With regards to the latter, interviewees were asked about their care practices but discussion with our data collectors led us to believe that their answers reflect knowledge of expected practices rather than actual staff behaviour and thus are best considered a proxy for knowledge. We aimed to interview the maternity in-charge or equivalent in each facility; this information therefore represents their knowledge. For the ‘infrastructure’ index, we used questions on the availability of, and access to key infrastructure and equipment in the maternity unit.

**Table 1.** Indices’ components by ‘clean’ and for each enabling factor

Enabling factor	Clean hands	Clean cord	Clean birthing surface
Knowledge and training	Wash hands during the WHO key moments of hand hygiene (no data on hand washing before aseptic procedures, so this was not included)	Frequency of use of sterile clamps or ties	Delivery room cleaned at least once a day
	AND	AND	AND
	Training on hand hygiene received in the last year	Training on IPC received in the last year	Training for non-medical staff received in the last year
Infrastructure	(1) Soap available in the maternity unit	(1) Disposable or sterile clamps available in the maternity unit	(1) Bleach or bleaching powder currently available
	AND	AND	AND
	(2) Disposable gloves available in the maternity unit	(2) Disposable or sterile blades available in the maternity unit	(2) Delivery bed available and functional
	AND	AND	AND
	(3) Water is improved and available (24h availability, AND functional sink AND available AND piped water supply is not interrupted more than once a week)	(3) If reusable equipment is used, any sterilization method (i.e. products for High-level Chemical Disinfection, autoclaves, autoclave, dry heat sterilizer or boilers) available and functional	(3) Water is improved and available (24 h availability, AND functional sink AND available AND piped water supply is not interrupted more than once a week)
Staffing levels	At least one SBA present during the morning and night shift prior to the survey	At least one SBA present during the morning and night shift prior to the survey	At least one orderly present during the morning shift prior to the survey
Policies or posters on	Hand washing	‘Not applicable as we did not collect this information’	Decontamination of areas contaminated with body fluids

For the ‘policies’ determinant, we present data on whether policies or posters of key protocols i.e. IPC, hand hygiene and decontamination of areas soiled by blood and other body fluids were available in the maternity unit. For ‘human resources’, at least one skilled SBA should be present in the maternity during the morning and night shifts; this ensures that someone formally trained in IPC is available on site capable of cleaning their hands adequately at appropriate times and capable of performing clean cord care. Since it was unusual in Zanzibar, especially in small facilities, that orderlies were allocated to night shifts, for clean birth surface the variable we referred to was whether an orderly was present on the previous morning shift.

The indices were all binary, with facilities either meeting all the conditions prescribed by the index or not. Similar composite indices have been used previously to describe key markers of the quality of maternal healthcare facilities (Nesbitt *et al.* 2013; Campbell *et al.* 2016). The key assumption was that the components chosen to construct the indices were fundamental for performing the ‘cleans’.

### Analysis

The variety of tools used produced quantitative, qualitative and microbiological data. Results from all three tool sets were organised thematically using the frameworks discussed: the WHO cleans and the enabling factors.

The water analysis, using conventional pour plate and membrane filtration techniques, focused on the total bacterial count in the water samples, as well as looking at the presence of *Enterococcus* and fecal coliforms—standard indicators for assessing water quality (Ashbolt *et al.* 2001). Swabs collected from surfaces were directly inoculated onto selective media and screened using standard biochemical techniques to identify and characterize potential pathogens. The analysis of the microbiology swab data focused first on whether *Staphylococcus aureus* (*S. aureus*), one of the most common pathogens linked to healthcare associated infections (Allegranzi *et al.* 2011), was present at the touch site. Opportunistic pathogens such as *S. aureus* are frequently shed by patients and staff in healthcare environments and can persist on surfaces for months on dry surfaces, posing a significant transmission risk to new patients admitted to the facility—thus, we used this as an indicator for cleanliness (Kramer *et al.* 2006). The second indicator examined was whether multiple pathogenic organisms were identified on the touch site. Two or more such pathogens found on a hand touch site indicate a lack of effective cleaning or long durations between cleans. For more details see Supplementary Material S1.

We began our analysis of the qualitative materials with word-for-word transcriptions of the audio files in their original language. Transcripts were later translated into English and analysed manually using a qualitative ‘content analysis’ method to extract manifest and latent content from the interviews (Vaismoradi *et al.* 2013). We used an inductive process for analysis whereby all codes and themes were derived from data. No software was used, a research assistant coded the data manually and the senior qualitative researcher reviewed the codes to check their quality (all codes are available on request).

Using facility questionnaire responses, indices representing each of the four enabling factors were constructed for each ‘clean’ and described by facility type. In our dataset, we distinguished between three types of facilities: with an operating theatre or without, and those which the MoH had not deemed appropriate to perform deliveries because they lacked key equipment and infrastructure. Since facility questionnaire data came from all facilities providing maternity services in Zanzibar, no survey weights were applied. The walkthrough checklist data produced counts of the infrastructure and equipment available, cleaned, and according to state of repair. Data were double entered into EpiData v3.1 and analysed using STATA v13 SE.

### Ethics approval and consent to participate

We obtained ethical approval from the Zanzibar Medical Research and Ethics Committee and the Observational/Interventions Research Ethics Committee at the London School of Hygiene and Tropical Medicine for this study. The women interviewed gave their individual consent, while the MoH granted permission to interview healthcare staff, and collect and analyse microbiology samples in the facilities.

Women who gave birth recently—respondents were informed about the purpose of the survey before the start of the interview, informed that their participation was voluntary, and that all information provided was confidential and would be de-identified. The respondent's consent, if obtained, was in written form.

Facility data—prior to commencing the facilities questionnaire, an official letter was sent by the MoH to all facilities to inform them of the study aims and that the information collected might be used by the MoH or other organizations seeking to improve the planning and delivery of health services, and that the identity of the facility would be anonymized. For each of the seven facilities selected for the semi-structured interviews and the walkthrough this information was also provided in person by the enumerator to the facility in-charge, the maternity in-charge and the orderlies in-charge.

## Results

**Figure 1.**
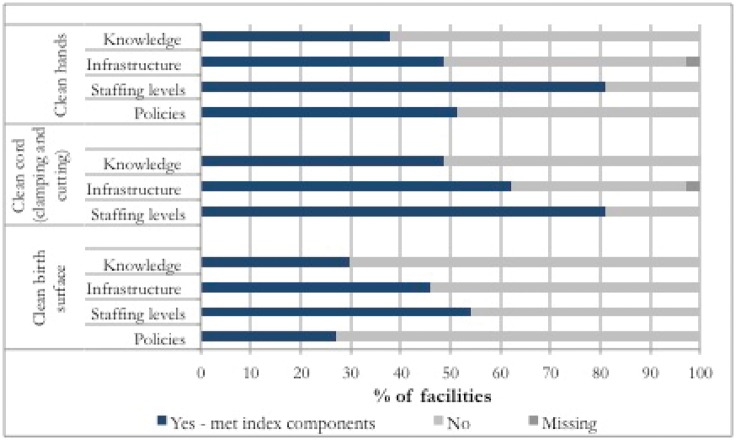
Percentage of facilities meeting all components per enabling factor index by clean (Knowledge stands for knowledge & training)

Of the 37 facilities providing childbirth services in Zanzibar, eight had an operating theatre, 24 did not, and five were considered by the MoH to be too poorly equipped to perform deliveries because of lack of water and delivery equipment. 84% of facility births across the 37 facilities surveyed took place at one of the eight facilities with an operating theatre (data not shown). The enabling factors’ indices for each of the ‘cleans’ were met by only 50% or fewer of the 37 facilities, with two exceptions: the infrastructure index for clean cord and the proportion of facilities with an SBA present in the morning and night shift before the survey, as described further below (Figure 1).

### Clean hands

Coverage of knowledge and training around clean hands was 38%, with 14 facilities out of the total of 37 meeting all the knowledge and training conditions (Table 2 and Figure 1). The weakest knowledge and training index component was knowledge around when to wash hands and, in particular, many respondents did not know they were supposed to wash hands ‘after touching the environment around the patient’. In 70% of facilities, staff reported having had training on hand hygiene, and this was confirmed by the qualitative interviews. Almost all care providers with which we conducted qualitative interviews could explain the hand hygiene process correctly (*n* = 26).

**Table 2.** Proportion^a^ of facilities meeting the enabling factors’ indices by ‘clean’ and facility type (data source: facility questionnaire)

Variable	Facilities with an operating theatre (*n* = 8) *n* (%)	Facilities without an operating theatre (*n* = 24) *n* (%)	Facilities deemed inappropriate for deliveries (*n* = 5) *n* (%)	Total facilities (*n* = 37) *n* (%)
Clean hands				
Knowledge and Training				
* Yes*	4 (50)	9 (38)	1 (20)	14 (38)
* No*	4 (50)	15 (63)	4 (80)	23 (62)
* Missing*	0	0	0	0
Infrastructure				
* Yes*	7 (88)	9 (38)	2 (40)	18 (49)
* No*	1 (12)	15 (63)	2 (40)	18 (49)
* Missing*	0	0	1 (20)	1 (3)
Staffing levels				
* Yes*	8 (100)	21 (88)	1 (20)	30 (81)
* No*	0	3 (13)	4 (80)	7 (19)
* Missing*	0	0	0	0
Policies				
* Yes*	6 (75)	12 (50)	1 (20)	19 (51)
* No*	2 (25)	12 (50)	4 (80)	18 (49)
* Missing*	0	0	0	0
Clean cord				
Knowledge and Training				
* Yes*	6 (75)	11 (46)	1 (20)	18 (49)
* No*	2 (25)	13 (54)	4 (80)	19 (51)
* Missing*	0	0	0	0
Infrastructure				
* Yes*	6 (75)	14 (58)	3 (60)	23 (62)
* No*	2 (25)	10 (42)	1 (20)	13 (35)
* Missing*	0	0	1 (20)	1 (3)
Staffing levels				
* Yes*	8 (100)	21 (88)	1 (20)	30 (81)
* No*	0	3 (13)	4 (80)	7 (19)
* Missing*	0	0	0	0
Clean birth surface				
Knowledge and Training				
* Yes*	5 (63)	4 (17)	2 (40)	11 (30)
* No*	3 (38)	20 (83)	3 (60)	26 (70)
* Missing*	0	0	0	0
Infrastructure				
* Yes*	6 (75)	9 (38)	2 (40)	17 (46)
* No*	2 (25)	15 (63)	3 (60)	20 (54)
* Missing*	0	0	0	0
Staffing levels				
* Yes*	8 (100)	11 (46)	1 (20)	20 (54)
* No*	0	13 (54)	4 (80)	17 (46)
* Missing*	0	0	0	0
Policies				
* Yes*	5 (63)	5 (21)	0	10 (27)
* No*	3 (38)	19 (79)	5 (100)	27 (73)
* Missing*	0	0	0	0

a The proportion was approximated to the nearest decimal; hence, variables options might not add up.

The facility questionnaire (*n* = 37) showed 18 facilities (49%) met all the infrastructure conditions for hand washing (Table 2 and Figure 1). The availability of a functional sink (i.e. a sink which can accommodate running water flowing from a tap) and whether running water is available 24 h a day were the main gaps in facilities’ hand washing infrastructure. Of the 22 hand-washing stations (including buckets and sinks) across 7 facilities surveyed in the walkthrough checklist, 15 had water available. When water was not available, facilities use stored water. Due to logistical difficulties in accessing the storage containers, we were only able to take samples from two water storage containers at two of the seven facilities: a plastic bucket and a larger plastic container. Both showed high levels of contamination; their total bacterial count was over 300 CFU/ml, and one sample had a high presence of *Enterococcus* (100 CFU/ml). We also took samples from water sources routinely used for hand washing, and 21% of these (*n* = 102) had a total bacterial count of over 100 CUF/ml (See details on the water analysis results in Supplementary Material S1). Indeed, 73% of the facilities surveyed reported water testing is never done in the facility, and the rest did not know this information. 

The qualitative interview analysis (*n* = 26) emphasized that water availability was a major challenge. A common substitute for the lack of piped water was to store water in buckets. At two facilities out of seven, staff reported having to carry water in buckets from water storage tanks outside the facility, due to blockages in pipes. Maintaining a sufficient water supply was an issue, particularly at night when institutional availability of water is less reliable and those in charge of maintenance are not on shift.

In 12% of the facilities without an operating theatre (*n* = 24), there was no SBA during the morning and night shift prior to the survey (Table 2); whereas, all facilities with an operating theatre had at least one SBA present. Staffing shortages and high caseloads were frequently mentioned during qualitative interviews as reasons for poor IPC.

The facility questionnaire (*n* = 37) data showed that policies or posters about hand washing were available in 51% of facilities (Table 2); this proportion was 75% for facilities with an operating theatre. The walkthrough revealed that only three of the seven maternity wards observed had a poster on hand hygiene displayed in the maternity area.

### Clean cord

From the facility questionnaire (*n* = 37), 18 facilities (49%) met the knowledge and training conditions and 23 facilities (62%) met the basic infrastructure conditions for a clean cord (Figure 1). All facilities reported routinely using disposable blades and cord clamps, but these were not always available; 89% of facilities had sterile blades available, but only 68% had both sterile cord clamps and sterile blades (data not shown). One facility reported commonly using reusable cord clamps but also reported having no functioning sterilization or high level disinfection equipment.

Walkthrough data showed similar results: all seven facilities had access to either reusable or disposable cord cutting equipment. The walkthrough supplemented the questionnaire findings by showing whether equipment for cord care was decontaminated (if reusable) and stored safely. Similar to the facility questionnaire results, access to cord clamps was lower than for blades. Qualitative interviewees at five of the seven facilities reported creating self-made cord ties from the rim of sterile gloves or pieces of string, ideally soaked in alcohol solution. Potential failure in carrying out this procedure makes strings less safe and practical than disposable sterile clamps.

The staffing levels for clean cord care were measured in the same way as for clean hands as reported above. We did not collect specific information on policies around clean cord.

### Clean birth surface

All the basic conditions for knowledge and training index around a clean birth surface were met by 11 out of 37 facilities (30%) (Table 2 and Figure 1). A weak component of index was the lack of training for non-medical staff, including orderlies, who are responsible for cleaning the bed surface.

The walkthrough checklist results confirm these findings. Microbiological samples revealed that in six of the seven facilities where swabs were taken, the maternity beds were highly contaminated with multiple organisms, especially around the perineal area. Sixty percent of mops and mop bucket swab sites tested positive for multiple microbiological organisms. Multiple organisms were further identified on six out of eight surface cleaning cloths. It was a common finding that most mops were stored inside buckets filled with mopping fluid for most of the day.

The infrastructure index suggests that only 17 out of 37 facilities (46%) met the basic requirements for a clean birth surface (Table 2), with the weakest index component being the same as for clean hands: consistent availability of water (Figure 1). The facility questionnaire (*n* = 37) found that all but two facilities surveyed had at least one functional delivery bed available (data not shown). The results from the walkthrough checklist found that in both the maternity and delivery rooms, most beds (21/26) across the seven facilities surveyed were covered in cleanable materials and/or a mackintosh (data not shown).

Across all seven facilities where qualitative interviews (*n* = 26) were conducted, staff complained about a shortage of orderlies. In line with these findings, the facility questionnaire (*n* = 37) revealed that only 54% of facilities had an orderly present in the maternity unit on the morning before the survey (Table 2). The shortage of orderlies was further aggravated by the fact that most of the orderlies interviewed also performed healthcare related tasks such as antenatal care, wound dressing, prescribing medications and assisting deliveries, which significantly reduced the time they spent on cleaning activities.

Of the facilities without an operating theatre, only 21% had policies or posters on the decontamination of areas contaminated with body fluids (Table 2). The proportion was higher for those facilities with an operating theatre, 63%.

## Discussion

We provided an illustrative analysis of IPC information collected in maternity units in a low-income country to assist in developing a quality improvement strategy both at the local facility and the MoH levels. Our results are actionable for three main reasons: the use of a clear framework, the WHO IPC guidelines, made up of four enabling factors amenable to change; the use of mixed methods to unpack the complex picture behind the infection prevention gaps; and the focus on and relevance to the key interventions necessary to reduce maternal and newborn infection embedded in the WHO clean practices: making sure that during labour and delivery the hands of the birth attendants, the birth surface and the cord clamping and cutting are all clean.

Using the WHO IPC guidelines framework we could organise our results so that the MoH could identify the weakest enabling factors of the necessary clean practices and the type of intervention needed—e.g. infrastructure vs training. For example: the weakest index component for clean birth surface was the knowledge of health orderlies and their lack of training on decontamination of areas exposed to body fluids. The theme of knowledge in itself helped narrow down the potential for action to an educational intervention involving specific roles in the MoH, such as district level supervisors and the local institute for nursing training.

To produce data on IPC gaps that can be actioned by the MoH required a mixed-methods approach to data collection and analysis. Our mixed methods approach provided a comprehensive and useful description of key enabling factors of the relevant clean practices in maternity units, with different methods suited to different items of information. For example, the facility questionnaire revealed that water is often unavailable on the labour ward. With this information alone we did not know whether delivery was practiced in the absence of running water or how the problem was overcome. Through semi-structured interviews, we learned that staff perform deliveries without running water, and that standing water buckets are used as an alternative to non-functioning sinks. Although very limited in number, the standing water buckets we sampled were highly contaminated; as found elsewhere, inappropriate water storage leads to contamination (Shields *et al.*; Wright *et al.* 2004). The triangulation of data strengthened our conclusions, and avoided some of the assumptions inherent in the interpretation quantitative results. The mixed methods approach allowed us to understand the complex picture behind the IPC weaknesses we found and to provide potential intervention targets to the ministerial audience.

Our approach to producing actionable information is unable to recommend which of the enabling factors will have a sustainable and wider benefit; indeed, it probably draws attention towards shorter-term solutions such as infrastructure and training that are quick wins for any MoH, compared to longer-term structural changes. Yet, our approach still highlights these wider structural gaps—such as the lack of sufficient staff and policy gaps.

Although no agreed definition for ‘actionable information’ exists in global health, other research using this terminology refers to information presented in a way that makes evidence-based programming more accessible, using for example the visual display of data (Makulec and Morgan 2015). This was also our intent and fits into the current wider attempt in public health to ensure that evidence feeds into action by using condition specific frameworks and platforms (Evidence for Action); Swinburn *et al.* 2005). Using a clear and simple approach to identify actionable information was an important ingredient for the project’s endorsement and support from the MoH; yet translating that information into action would not have been possible without a participatory workshop that included all key stakeholders. We describe how we engaged with the key stakeholders in a participatory workshop and how the information presented was then translated into action in Supplementary Material S2.

An important limitation to our actionable information approach is that we looked at proxies of the enabling factors rather than actual practices. Ideally, both should be done, but time and financial limitations meant that we could not observe practices. We would also have liked to explore more enabling factors, but the type of data we collected did not permit this. In particular, the tools we used did not collect information on social norms and individuals’ motivation—key areas for explaining behaviour (Montano and Kasprzyk 2008).

The results show that overall facilities’ performance across all enabling factors for each of the ‘cleans’ was poor. Each enabling factors’ index was met by, at best, half of facilities, apart from two factors met by a higher proportion. However, even these better performing indices are of concern. Only 81% of facilities had SBAs present in the morning and night shift before the survey; a finding supported by the low presence of skilled personnel in maternity wards in Eastern African shown by a recent multi-country study (Kruk *et al.* 2016). Indeed, this index should be at 100% as facilities providing maternity care should run with 24 h services. In this context, in the absence of an SBA, deliveries are occasionally performed by health orderlies. Across virtually all indices, facilities with an operating theatre performed better, in terms of knowledge, infrastructure, availability of staffing and policies, compared with smaller facilities providing basic obstetric care. This is consistent with other studies showing that larger facilities generally tend to score better in terms of some markers of quality of care (Campbell *et al.* 2016).

Other key findings included first, the substantial lack of a reliable and constant water supply, with half of facilities operating without basic water infrastructure. This is consistent with research on water availability in facilities in low- and middle-income countries (LMICs) (Chawla *et al.* 2016) and specifically in maternities in Tanzania (Benova *et al.* 2014; Gon *et al.* 2016). A recent review (Bain *et al.* 2014) of water quality in LMICs found very few studies based in health facilities, highlighting the importance of our data in this field. They proposed a score to assess the quality of water sampling and analysis. Applying their system, our study met 10 out of 13 quality criteria, which is above the interquartile range of the 319 studies in their review (Bain *et al.* 2014).

Another key finding was the poor knowledge and training and practice of health orderlies in cleaning the birth surface—from the walkthrough exercise we found that six of the seven maternity units swabbed had beds with *S. aureus*, representing a lack of effective or frequent cleaning. A very recent study in paediatric wards with poor cleaning practices in South Africa also found *S. aureus* on their surfaces (Dramowski *et al.* 2016). A study from India which includes the maternity unit environment, found that 10% of patient care equipment was contaminated with some kind of pathogen (Dadhich *et al.* 2014). In addition, the facility questionnaire reported that 37% of facilities cleaned the delivery room less than once a day on average and their non-medical staff were un-trained. The high levels of pathogens present on the cleaning equipment may explain the high level of microbiological contamination found on the beds. Overall cleaning in healthcare facilities is a poorly monitored and an under-researched area in spite of being vital to effective IPC and the reduction of healthcare associated infection. Simple solutions like fluorescent gel and UV markers can promote local engagement and training of cleaners (Dramowski *et al.* 2016).

We have confidence in our results given the consistency across the different tools used and because indices were constructed using data from all maternity units across Zanzibar. Moreover, our findings were consistent with the views on the status of IPC in maternities expressed by workshop participants including the MoH. Results of the enabling factors’ indices, should, however, be interpreted cautiously, especially for knowledge and training of staff which was based on the response of only one person at each facility. Having said this, as we aimed to interview the maternity in-charge, or equivalent at the time, at each facility, we expect the results are fairly representative of the maternity unit personnel. If anything, our choice of interviewee may overestimate the average knowledge of the personnel in the maternity unit. With regards to the staffing indices, having at least one SBA or health orderly available does not guarantee clean practice – but their presence would increase the likelihood of the ‘clean’ being performed. As mentioned earlier, in the absence of an SBA, deliveries are occasionally performed by health orderlies with no formal training in delivering a baby including relevant aspects of IPC.

These data may be influenced by observer bias because the data collectors were MoH employees for all tools except the semi-structured interviews. However, two things minimise this issue: first, data collectors were sensitised repeatedly about the fact that data were collected mainly for local improvement purposes and needed to be accurate for this to be possible. In addition, we emphasised that data would be anonymized, so there should be no repercussions for interviewees, facilities, or interviewers. Second, the walkthrough tool set and the semi-structured interviews at each of the seven facilities were closely supervised by an independent senior qualitative scientist. The results from these tools were consistent with the facility questionnaire results, providing further evidence that observer bias might not have influenced our results significantly.

Quantitative analysis of environmental samples was not possible due to limited laboratory capacity, although 30% of the swabs yielded levels of growth too high for quantification. Indeed, this was the first time, the Pemba Health Laboratory carried out environmental sampling and analysis. Not many healthcare laboratories in low-income settings have exposure to environmental sampling and therefore greater advocacy, training and support for laboratories would lead to standardization of swabbing techniques, sample culturing and reporting.

A further limitation is that information on the availability of electricity which is key to performing a clean delivery, especially at night, was not collected (Adams *et al.* 2008). From the 2014 Service Provision Assessment of healthcare facilities, we know that 77% of facilities have regular electricity in Zanzibar (Tanzania Service Provision Assessment Survey 2014-2015, 2016). Other information related to infection prevention during birth was collected, such as on waste disposal, and availability of malaria bed nets; however, this is not presented as it does not directly relate to our outcome framework.

We present a simple approach to analysing IPC data from maternity units to facilitate and prompt action. Using our approach, the Zanzibar MoH was able to readily prioritise and follow-up on the findings presented here by organising for the first time a formal training for health orderlies on cleaning practices, and by improving the infrastructure of sinks in the maternity wards. Observation of the actual clean practices would significantly improve our approach but could be prone to a non-trivial Hawthorne effect. Using this approach in other settings/countries could provide key evidence for governments to improve maternity units, and so contribute to the prevention of newborn and puerperal sepsis.

## Supplementary Material

Supplementary File 1

Supplementary File 2
