# Supplementary material for: Unpacking the enabling factors for hand, cord and birth-surface hygiene in Zanzibar maternity units
Source: Health Policy Plan. 2017 Jul 12;32(8):1220–8. doi: 10.1093/heapol/czx081 (PMC5886267; doi:10.1093/heapol/czx081)
Supplement: Supplementary File 1 [file supplementary_file_1_czx081.docx]

# Supplementary File 1

## Surfaces and water sampling

Trained personnel supervised surface and water sample collection and laboratory technicians at an accredited laboratory analyzed samples according to local standard operating and quality control procedures.

Swab samples from selected surface areas were collected by the data collection team during facility walkthroughs. To take surface swabs the data collection team soaked the tip of a sterile swab with sterile phosphate buffered saline. The selected area was swiped using the dampened swab (for flat surfaces a sterile template –(10x10cm) was used, for irregular surfaces e.g. door handles & taps, the entire surface area was swabbed). Swabs were taken by applying even pressure and rotating the swab for 30 seconds at each selected area. The swabs were transferred aseptically into the transport media and were labelled with the corresponding unique photograph number. Samples were placed in a cool box (at 4 - 8◦C) and transported to the laboratory for analysis.

Water samples (500 ml volumes) were collected from all available water sources (both improved and unimproved) in maternity units for each of the seven facilities, according to standard operating procedures. An assessment was made regarding the environmental condition of each water source (for example, signs of leakage, standard of cleanliness). When samples were collected from a tap; the tap was sanitized with 70% alcohol and water flushed through for 60 seconds prior to sample collection. Samples from water storage containers were collected using sterile cups and then transferred to sterile glass bottles. Water samples were transported to the laboratory within two hours of collection and were stored between 2 ᵒC – 10 ᵒC for the duration. Samples were processed upon arrival at the laboratory. No chemicals were used t to neutralize residual disinfectants potentially present in water samples; 30% of facilities reported that water was untreated and a further 8% of facilities were unaware of treatment.

## Microbiological analysis

Water Samples

Using conventional pour plate and membrane filtration techniques, analysis of water samples focused on total bacterial count, and the presence of *Enterococcus* and fecal coliforms respectively, as standard indicators used to assess water quality. Only single water samples were analyzed – no duplicates were collected. Neither pH, nor turbidity testing was carried out.

Surface Samples

The analysis of the environmental swab data focused on two standard indicators of microbiological cleanliness. First, whether or not the swab site had *Staphylococcus aureus* (*S. aureus*) present. Opportunistic pathogens such as *S. aureus* are frequently shed by patients and staff in health care environments and can persist on surfaces for days, posing a significant transmission risk for new patients admitted to the facility. *Staphylococcus* isolates were presented as either coagulase-positive or coagulase-negative. *S. aureus* is coagulase-positive and is regarded as the most medically significant species of the genus; as such it is one of the most common pathogens linked to healthcare associated infections. We therefore focused on the presence or absence of *S. aureus* as an indicator of cleanliness. At the time of swab collection and analysis, the laboratory lacked the capacity to screen for antibiotic resistance. The second indicator examined was the presence or absence of multiple pathogenic organisms on the swab site. Further to *S. aureus*, each opportunistic pathogen poses a clinical risk, some to a greater extent than others. If two or more such pathogens are found on a hand-touch site it indicates a lack of effective cleaning or long durations between cleans.

## Water analysis results - frequency of different CFU levels of bacteria, enterococcus and fecal coliform in hand-washing water

|  | 0 | 1-10 | 11-100 | 101-300 | 300+ | TOTAL |
| --- | --- | --- | --- | --- | --- | --- |
| B.Count | 0 | 0 | 13 | 17 | 4 | 34 |
| E | 11 | 10 | 13 | 0 | 0 | 34 |
| F.C | 26 | 8 | 0 | 0 | 0 | 34 |
| TOTAL | 37 | 18 | 26 | 17 | 4 | 102 |
