# Supplementary material for: Unpacking the enabling factors for hand, cord and birth-surface hygiene in Zanzibar maternity units
Source: Health Policy Plan. 2017 Jul 12;32(8):1220–8. doi: 10.1093/heapol/czx081 (PMC5886267; doi:10.1093/heapol/czx081)
Supplement: Supplementary File 2 [file supplementary_file_2_czx081.docx]

# Supplementary File 2

We describe here how we engaged key stakeholders in a participatory workshop, which enabled us to translate this information into action. The findings were presented on the first day of a three-day participatory workshop held in November 2014 with the MoH and the project partners and relevant stakeholders such as the Zanzibar College of Health Sciences, Jhpiego and the Zanzibar Water Authority. The workshop was used to a) finalise priorities among the findings and b) develop action plans to tackle those agreed priorities. A debrief between the key workshop organisers identified the following key features to be the main reason for the success of the workshop: first, an action-oriented focus was a priority from the beginning of the project. Well established participatory quality improvement exercises were used to prioritise and develop action plans; these were the “fish bone exercise” and “plan, do, study and act” cycles respectively(NHS Institute for Innovation and Improvement, 2008a, 2008b). Second, ahead of the workshop, a smaller workshop with a few key stakeholders was held in October 2014, to gather feedback on the way information should be presented to the group. From this initial meeting, it was decided to use pictures, microbiology findings and text as the informational channels. Graphs were not used prominently. Third, a good range of key decision makers at national, district and facility level; and representing both the government and the key partners, were engaged in the process to ensure ownership and collaboration at all levels.

The priorities identified during the workshop were a lack of good practices and training for health orderlies and the limited availability of functional sinks on the maternity units. The workshop participants agreed on the following action plans for 2015-2016:

- **Training for all health orderlies on waste management, cleaning techniques and cleaning and maintaining equipment.** The training curriculum for this was developed by the Zanzibar MoH and 30 orderlies were trained. Currently the training curriculum is being rolled out in Tanzania mainland.
- **Ensure that at least one functional sink is available in each maternity unit.** In collaboration with WaterAid Tanzania, the Zanzibar MoH developed a staggered implementation plan to accomplish this across nine facilities where no functional sink was available.

## Supplementary File 2 references

NHS Institute for Innovation and Improvement. 2008a. Plan, Do , Study, Act (PDSA).

NHS Institute for Innovation and Improvement. 2008b. Cause and Effect (Fishbone).
